# Biochemical and immunological parameters as indicators of osteoarthritis subjects: role of OH-collagen in auto-antibodies generation

**DOI:** 10.17179/excli2014-423

**Published:** 2015-09-23

**Authors:** Jalaluddin M. Ashraf, Quazi S. Haque, Shams Tabrez, Inho Choi, Saheem Ahmad

**Affiliations:** 1 School of Biotechnology, Yeungnam University, Gyeongsan, Republic of Korea; 2 Department of Biochemistry, Hind Institute of Medical Sciences, Barabanki, U.P., India; 3 King Fahd Medical Research Center, King Abdulaziz University, Jeddah, Saudi Arabia; 4 Department of BioSciences, Integral University, Lucknow, India

**Keywords:** osteoarthritis, collagen-II, biochemical and immunological parameters

## Abstract

Osteoarthritis (OA) is characterized by inflammation of the knee joint, which is caused by accumulation of cytokines and C-reactive protein (CRP) in the extracellular matrix as an early immune response to infection. The articular cartilage destruction is discernible by elevated tumour necrosis factor-α (TNF-α). In this study, blood samples of knee osteoarthritis patients were analyzed for biochemical and physiological parameters based on the lipid profile, uric acid, total leukocyte count (TLC), hemoglobin percentage (Hb%) and absolute lymphocyte count (ALC). Furthermore, immunological parameters including TNF-α , interleukin-6 (IL-6) and CRP were analyzed. The presence of antibodies against hydroxyl radical modified collagen-II (^•^OH-collagen-II) was also investigated in arthritis patients using direct binding ELISA. The uric acid and lipid profiles changed extensively. Specifically, increased uric acid levels were associated with OA in both genders, as were enhanced immunological parameters. The TNF-α level also increased in both genders suffering from OA. Finally, auto-antibodies against OH-collagen II antigen were found in the sera of arthritis patients. These results indicated that immunological parameters are better predictors or indexes for diagnosis of OA than biochemical parameters.

## Introduction

Osteoarthritis (OA) is a common, age-related, heterogeneous group of disorders characterized pathologically by focal areas of loss of cartilage in synovial joints that is associated with varying degrees of osteophytic formation, subchondral bone change which grip the whole joint, i.e. articular cartilage, subchondral bone alterations and joint-lining synovial membrane. Correspondingly, development of osteoarthritis involves elaborate interactions of cartilaginous tissue metabolism and maintenance, osteogenesis, mineralization and inflammation of the synovial membrane. Identification of the molecular pathways and individual factors involved in its etiology and understanding of mechanisms of their action and interaction are prerequisite to the development of accurate diagnostic and prognostic tools and providing OA patient's effective treatment. There has been great progress in understanding molecular mechanisms of OA appearance and progression, including identification of a network of biochemical factors that are important to normal functioning of the joints and changes leading to osteoarthritis (Livshits et al., 2010[21]). Despite these advances in knowledge regarding the biochemistry of OA, valid breakthroughs in this area are still needed.

There are also distortions in the synovial membrane and para-articular structures. Evidence has shown that biochemical and immunological parameters are important factors contributing to the development of OA. For example, estrogen deficiency has been reported to increase TNF-α production by monocyte-enriched peripheral blood mononuclear cells, as well as unfractionated human and murine bone marrow cells. Further, studies in humans have shown that adherent mononuclear blood cells contain CD3+ and CD56+ lymphocytes, a TNF-α producing subset of adherent T cells, and that the levels of these T cells decrease in response to estrogen treatment and are inversely correlated with bone density (Dantas and Sandberg, 2005[19]). Non-enzymatic glycation reaction has also been reported to play a role in the initiation and progression of arthritis (Saudek and Kay, 2003[27]). Non-enzymatic glycation of proteins, nucleic acids and lipids results in the formation of advanced glycation end products (AGEs) (Ahmad et al., 2013[3][8]; Akhter et al., 2013[10][12]; Rahim et al., 2014[25]; Shahab et al., 2014[32]; Ashraf et al., 2015[16][14][17]), as well as proteins such as collagen. AGEs cause pathologic stiffening of cartilage and extracellular matrix and accumulate with age. Pentosidine, an AGE, is present in serum, synovial fluid, and articular cartilage from patients with osteoarthritis (Mustafa et al., 2012[24]; Ashraf et al., 2014[15], 2015[14]). AGE modification of normal articular cartilage increases stiffness, facilitates chondrocyte-mediated proteoglycan degradation, reduces susceptibility to matrix metalloproteinase-mediated degradation, and decreases proteoglycan synthesis by chondrocytes. These observations parallel findings for osteoarthritic cartilage, suggesting that AGE modification could contribute to the pathogenesis of osteoarthritis (Saudek and Kay, 2003[27]).

OA is one of the most common joint disorders, and is more prevalent in females than in males. OA is primarily a non-inflammatory disorder of movable joints characterized by an imbalance between the synthesis and degradation of articular cartilage, leading to classic pathological changes of wearing away and destruction of cartilage (Mishra et al., 2013[22]). Many factors affect osteoarthritis, including age, gender, genetic factors, estrogen in women, exercise and sports (Roman-Blas et al., 2009[26]). The major structural protein of cartilage is type II collagen (Shahab et al., 2012[30]), which maintains and binds matrix molecules important for cartilage stability (IX and XI) (Creamer and Hochberg, 1997[18]). The cartilage stabilities are also affected by various cytokines, including interleukin-1 (IL-1) and TNF-α.

Bone resorbing activity of peripheral blood monocyte culture supernatants was shown to be higher in postmenopausal women than premenopausal women. Decreased blood estrogen levels and increased bone resorbing activity in postmenopausal women leads to IL-1 and TNF-α production from peripheral blood monocytes and bone marrow cells. IL-1 and TNF-α stimulate chondrocytes to produce more degrading enzymes and other cytokines such as IL-6, IL-8, nitric oxide and prostaglandin E2. 

The present study was conducted to identify physiological, biochemical and immunological factors playing a major role in the development of osteoarthritis disease in elderly patients. Furthermore, the presence of auto-antibodies was probed against native and hydroxyl-modified-collagen in the sera of OA patients.

## Materials and Methods

Anti-human alkaline phosphatase conjugate, p-nitrophenyl phosphate, Tween-20, protein-A agarose, sodium azide, and dialysis tubing were purchased from Sigma Chemical Company, USA. Flat bottom polysorp enzyme linked immunosorbent assay (ELISA) modules were acquired from NUNC, Denmark. All other reagents/chemicals were of the highest analytical grade available.

The present study was carried out on 100 human subjects aged 40-60 years, among which 20 were normal healthy controls and 80 were OA patients. These patients were taken from the outpatient department (OPD) of the orthopaedic department of Hind Institute of Medical Sciences (HIMS), Barabanki, India. Informed consent was obtained from all patients enrolled in this study. Additionally, the study was conducted according to the guidelines of the declaration of Helsinki and all the authors confirm that they have complied with the World Medical Association Declaration of Helsinki regarding ethical conduct of research involving human subjects.

### Collection of blood sample

10 ml of blood sample was taken from an ante-cubital vein under aseptic conditions. 2 ml blood was transferred to a citrate vial for erythrocyte sedimentation rate (ESR) estimation and 1 ml blood was transferred to an EDTA vial for physiological parameters and 7 ml blood was transferred to a plain vial for serum collection for immunological analyses. The blood sample, collected in a plain vial was kept for 30 minutes. Serum was separated from clotted blood, centrifuged at 3000 rpm for 5 min. Thus serum was kept at 56 °C for 30 min for de-complementation.

### Physiological parameters

Hemoglobin (Hb) was estimated using a Cayman's Hb assay kit (Schechter, 2008[28]). Briefly, blood was collected in a heparin vial and diluted with the provided sample buffer. A dilution of ≥ 1:10 was required before assaying a sample. Next, 200 µl of standard Hb was added to the standard wells of the plate. A total of 20 µl of blood and 180 µl of hemoglobin detector were then added in duplicate to the sample wells, after which the plate was covered with the plate cover and incubated for 15 minutes at room temperature. Finally, readings were taken at wavelength of 580 nm. 

The Erythrocyte sedimentation rate was measured (to detect inflammation associated with conditions such as infections, and autoimmune diseases) and evaluated by Fahraeus and Westergren method. In brief, 0.4 ml of 3.8 % sodium citrate (anticoagulant) was taken into 2 ml sterile syringe. Now fill the syringe up to 2 ml volume with venous blood from the cubital vein. Blow out the blood carefully from the syringe into the bowl. Press the pipette at the bottom of the screw and rotating the screw push the blood up to the mark 0. Start to measure the time. Tilt the pipettes with blood into 45° angle using a stand for pipettes. After 15 minutes read a value of ESR, which is approximately corresponding with the value read after 1 hour of investigation by classical method. 

### Biochemical parameters

Estimation of serum estrogen (Estradiol-17β-Estradiol, secreted by premenopausal ovary) was done according to the manufacturer, CAYMAN Chemical instructions kit. Estimation of uric acid (PAP-Uricase method) was done as per SRL diagnostic's instructions. Briefly, as described by Praetorius and Poulson, this method utilizes the enzyme uricase to oxidise uric acid. Pipette into test tubes labelled blank (B), standard (S), and test (T) as follows:

(Tab. 1)

Mix and incubate for 5 minutes at 37 °C at room temperature. Mix and read the absorbance at 546 nm.

(Tab. 2)

Estimation of serum cholesterol (CHOD-PAP method) was done as per SRL diagnostic's instructions. Pipette into test tubes labelled blank (B), standard (S), and test (T). Mix, incubate samples for 5 min at 20-25 °C. Measure the absorbance of specimen (A_specimen_) and standard (A_standard_) against reagent blank. The colour is stable for 60 minutes. The intensity of the colour produced is directly proportional to cholesterol concentration. It is determined by measuring the increase in absorbance at 500-550 nm.

Cholesterol concentration was calculated by using the following formula:


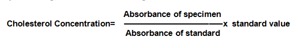


Estimation of serum triglycerides (GOP-ESPAS method), lipo-protein, and high density lipoprotein-cholesterol (HDL-C) (CHOD-PAP method) was done as per SRL diagnostic's instructions.

(Tab. 3)

50 µL of reaction mixture was added into each well. Mixing and incubation at room temperature for 60 minutes was done. Absorbance was taken at 570 nm. Mean absorbance blank value was subtracted from all the standard and sample readings. Finally standard graph was plotted to calculate triglyceride.

(Tab. 4)

Mixing of reagents for the number of assays was done. 50 µL of total cholesterol reaction mixture was added into the standard wells, as well as into the total cholesterol sample wells. Furthermore, 50 µL of free cholesterol reaction mixture was added to free cholesterol sample wells, mixed and incubated at 37 °C for 60 min. Absorbance was taken at 570 nm. Calculation was done by subtracting the mean absorbance value of the blank from all standard and sample readings. Finally, standard graph was plotted to calculate cholesterol (LDL-C/HDL-C) concentration.

### Immunological parameters

*Determination of C-reactive protein (CRP): *CRP estimation was done as per Cayman Chemical instructions. In brief, addition of 50 µL of sample diluent (contain 0.1 % sodium azide) was added into 96 well plate. After sample diluent, 50 µL of standard or sample was added to wells in duplicate. Plate was covered and incubated at room temperature (RT) for 1 hour. Plate was washed three times and 100 µL of Biotinylated Antibody Reagent (contains 0.1 % sodium azide) was added to wells and incubated at RT for 1 hour. Plate was further washed for three times to remove the unbound antigen. 100 µL of Streptavidin HRP (horseradish peroxidase) reagent was added to each well and plate was incubated at RT for 30 minutes. Plate was again washed for three times and addition of 100 µL TMB (3,3′,5,5′-Tetramethylbenzidine) substrate was done to each well and incubated in the dark for 30 minutes. 100 µL of Stop Solution was added to each well and absorbance was measured at 450 and 550 nm. Generation of standard curve was done by plotting the average absorbance (450 nm minus 550 nm) for each standard concentration on the vertical (Y) axis vs the corresponding TNF-α concentration on the horizontal (X) axis.

*Determination of TNF-*α*:* Estimation of TNF-α was done as per Life Technologies instructions: In brief, empty wells were rinsed upto four times by diluted assay buffer. Each well was completely filled with assay buffer during each wash. The plate was inverted between washing to empty the fluid from the wells. After the last washing, gently tap the inverted plate on absorbent to remove assay buffer. Now, addition of 100 µl of CRP TMB substrate solution to each well was done. The plate was covered with plastic film and incubated for 15 minutes at RT in the dark. Optimum development was obtained by using an orbital shaker equipped with a large, flat cover to allow the plate(s) to develop in the dark. Addition of 100 µl of CRP HRP stop solution to each well was done. Blue colour turned into yellow and colourless wells remained colourless; absorbance was taken at a wavelength of 450 nm.

*Estimation of Rheumatoid arthritic factor:* Rheumatoid factor (RF) estimation was done as per Life Technologies instructions. This assay employs the qualitative enzyme immunoassay technique. The microtiter plate provided in the kit was pre-coated with antigen. Samples (50-100 µl) were pipetted into the wells with anti-human IgA conjugated horseradish peroxidase (HRP). Antibodies, specific for the present antigen was bound to the pre-coated antigen. Now, plates were washed to remove any unbound reagent, a substrate solution was added to the wells. Colour was developed in proportion to the amount of human RF antibody (IgA) bound in the initial step. The colour development was stopped and the intensity of the colour was measured. Detection wavelength: 450 nm versus 620 nm within 30 min after adding the stop solution. Result was calculated by standard curve plot.

*Estimation of IL-6:* IL-6 estimation was done as per Life Technologies instructions. In brief, 100 μL of the Standard Diluent Buffer was added to zero wells. Well(s) reserved for chromogen blank was left empty. 100 μL of standards, samples or controls were added to the appropriate microtiter wells. Pipetted 50 μL of biotinylated anti-IL-6 (Biotin Conjugate) solution was pipetted into each well except the chromogen blank(s), incubated for 2 hours at RT. Wells were washed 4 times and addition of 100 μL Streptavidin-HRP working solution was added to each well except the chromogen blank(s), incubated for 30 minutes at RT and wells were again washed for 4 times. 100 μL of Stabilized Chromogen was then added to each well. The liquid in the wells turned to blue. Addition of 100 μL of Stop Solution to each well was done. The absorbance was measured at 450 nm. Estimation of IL-6 concentrations for unknown samples and controls was done from the standard curve plotted.

### Detection of auto-antibodies against hydroxyl radical damaged collagen-II

The presence of antibodies against OH-collagen II in the sera of OA patients was evaluated by enzyme linked immune sorbent assays (Shahab et al., 2012[31]; Ahmad et al., 2014[7], 2011[1]). The blood samples were allowed to clot and sera were separated. The serum samples from normal and healthy people served as control. Briefly, microtitre wells were coated with one hundred microlitre of OH-collagen II (10 µg/ml in TBS, pH 7.4) and incubated for 2 hours at 37 °C and overnight at 4 °C, respectively. Each sample was coated in duplicate and half of the plate, devoid of antigen, served as control. The test-plate wells were emptied and washed thrice with TBS-T to remove the unbound antigen. Unoccupied sites were blocked with 150 µl of 1.5 % non-fat dry milk in TBS (pH 7.4) for 4-5 hours at 4 °C followed by single wash with TBS-T. In direct binding ELISA, antibodies were directly added into antigen-coated wells and incubated for 2 hr at 37 °C and overnight at 4 °C, respectively. The wells were emptied and washed thoroughly with TBS-T. Anti-immunoglobulin G (Anti-IgG) alkaline phosphatase conjugate was added to each well and incubated at 37 °C for 2 hours and then the plates were washed thrice with TBS-T followed by a single wash with distilled water. Para-nitrophenyl phosphate was added and the developed colour was read at 410 nm on a microplate reader. The results were expressed as mean difference of absorbance values in test and control wells (A_test_ - A_control_).

## Results

Biochemical and physiological parameters analyzed are given in Table 5(Tab. 5). Among physiological parameters, Hb % did not change in male OA patients as compared to female subjects. The level of ESR was significantly increased in both male and female subjects of OA patients. Other parameters like TLC was found to be significantly decreased (*P<0.001; females **P≤0.05; males). The change in ALC was found to be negligible, indicating the occurrence of infection and inflammation in OA patients.

In biochemical tests, uric acid (UA) level was higher in both male and female OA patients. In lipid profile, total cholesterol (TC) and low density lipoprotein cholesterol (LDL-C) were significantly increased (P<0.001), while HDL-C was significantly decreased in OA patients of female subjects only. Triglyceride (TG) remained constant in both sexes, suggesting no role in causing OA. Moreover, the serum estradiol level was significantly decreased in female OA patients.

The immunological parameters estimated are given in Table 6(Tab. 6). Among immunological parameters, the percentage of CRP, TNF-α and IL-6 were significantly increased in male subjects (*P<0.001). This indicates induction of bone resorbing activity or tissue breakdown in OA patients. In female subjects CRP, TNF-α and IL-6 were also increased significantly (*P<0.001) (Table 6(Tab. 6)), which suggests that decreasing level of estradiol could be a cause of OA in females. 

To analyze the role of OH-collagen II in eliciting immune response in OA patients, the presence of auto-antibodies in arthritis subjects against OH-collagen II was probed by direct binding ELISA (Shahab et al., 2013[29]; Ahmad et al., 2011[5]; Moinuddin et al., 2014[23]; Akhter et al., 2014[11][13]). Out of 100 arthritic sera (50 male and female each), 30 male patient samples (60 %) showed higher binding with the OH-collagen II as compared to the native form, however, 40 female subjects (80 %) showed enhanced binding. These results point towards that most of the OA subjects (70 %) showed the presence of auto-antibodies against OH-collagen II suggesting the role of hydroxyl radical damaged collagen II in the OA patients. Type II collagen posttranslationally modified by OH**^.^** radical that are present in the inflamed joint is an autoantigen in OA patients.

## Discussion

OA is a destruction of cartilage bone with the period of time and collagen type II is one of the most pronounced long lived proteins responsible for it (Shahab et al., 2012[30]). Our results demonstrate that among all the parameters like physiological, biochemical and immunological, the immunological parameters showed intense changes or significantly induced level of TNF-α and IL-6 in both male and female patients suffering from OA. These swift in the levels of TNF-α and IL-6 indicates its most probable role in OA. In female subjects this might be due to the decrease in the level of estradiol, which decreases post menopause. Moreover, for the early detection of OA, the auto-antibodies (70 %) were found in the sera of patients against OH-collagen II. This points towards that there is the significant role of the hydroxyl radical in structural perturbations of the collagen II making it immunogenic.

Our research group is also working on some medicinal plants and its purified bioactive compounds having highly antioxidant property likes *Phullanthus virgatus* (Hashim et al., 2013[20]) and *Boerhaavia diffusa* (Akhter et al., 2013[10]). A research update on mechanistic approaches of these plants has also been discussed. Eventually, based on these plants antioxidant characteristics it is hypothesized that; the damage caused to the cartilage of OA patients can be recovered from bioactive compounds or plant extracts having potent anti-oxidant property. We have shown recently the antioxidant potential of some drugs and nanoparticles which might be used for the inhibitory activity of oxidative and glycative stress as well (Ahmad et al., 2013[3][8], 2014[4][6]; Ashraf et al., 2015[16][14][17]; Ahmad, 2014[2]; Ahmad and Siddiqui, 2015[9]). The anti-glycation and antioxidant potential of glycation assisted gold nano-particles (Gnps) have been recently discussed in bone cancer cells (Rahim et al., 2014[25]).

## Conclusion

It is concluded that immunological parameters are better forecaster for the diagnosis of OA than biochemical and physiological parameters. A defect of the immunological parameters might be use as foremost factors and mechanisms of compensation. The incidence of adverse effects for herbal medicines appears to be low and they may offer a much needed alternative for patients with OA. This study also opens an opportunity to study the effect of Gnps on arthritis as well.

## Notes

Jalaluddin M. Ashraf and Quazi S. Haque contributed equally to this work.

Inho Choi and Saheem Ahmad (Department of BioSciences, Integral University, Lucknow, India; e-mail: ahmadsaheem@gmail.com; saheem@iul.ac.in) contributed equally as corresponding authors.

## Acknowledgement

This research received no specific grant from any funding agency in the public, commercial or not-for-profit sectors.

## Conflict of interest

The authors declare that they have no competing interests.

## Figures and Tables

**Table 1:**
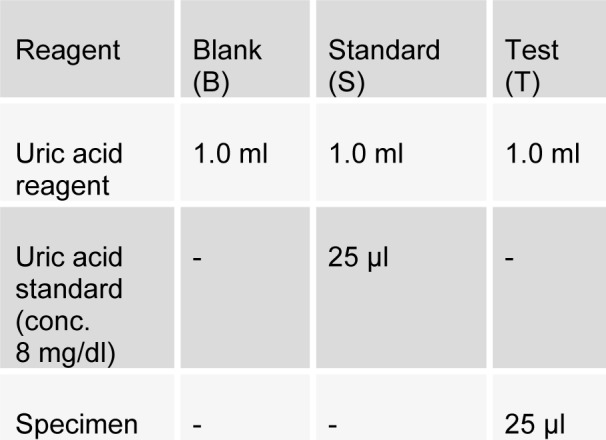
Biochemical parameters

**Table 2:**
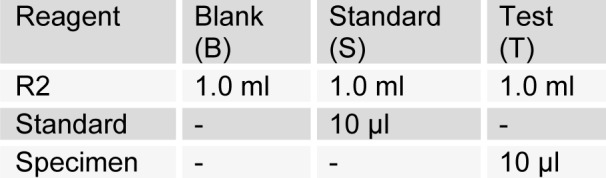
Serum cholesterol estimation

**Table 3:**
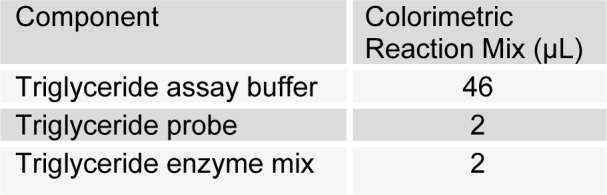
Serum triglyceride estimation

**Table 4:**
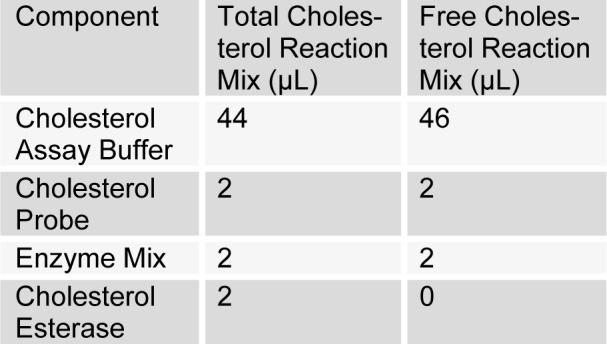
Lipoprotein and HDL-C cholesterol estimation

**Table 5:**
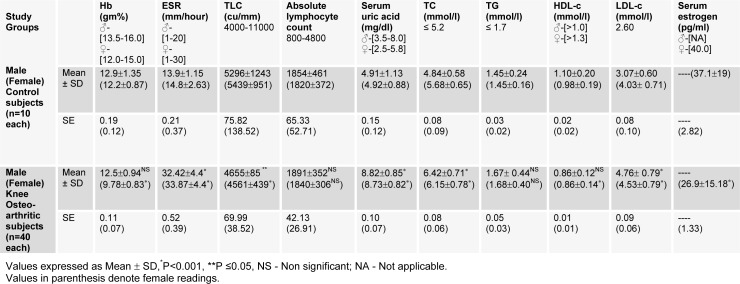
Status of physiological and biochemical parameters of male and female subjects of control and osteoarthritic groups

**Table 6:**
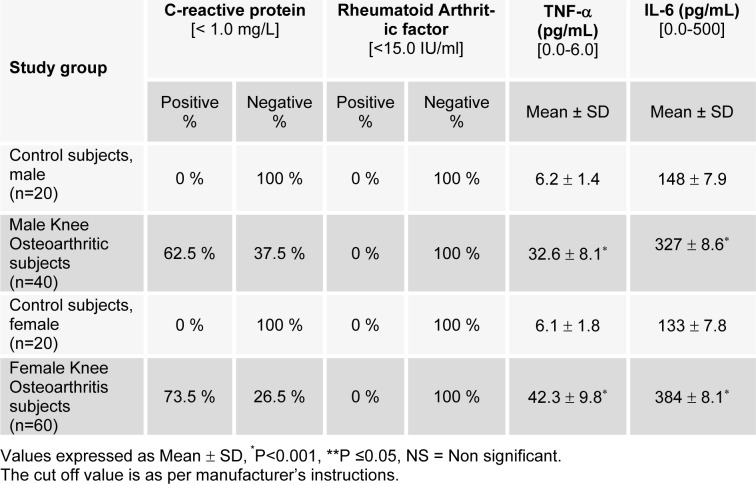
Status of immunological parameters of male and female subjects of control and osteoarthritic groups
